# Occupational adjustments and work ability of young adult cancer survivors: results from the AYA-Leipzig study

**DOI:** 10.1007/s00432-024-06050-4

**Published:** 2024-12-26

**Authors:** Hannah Brock, Katharina Schröter, Michael Friedrich, Annekathrin Sender, Diana Richter, Anja Mehnert-Theuerkauf, Kristina Geue, Katja Leuteritz

**Affiliations:** 1 Department of Medical Psychology and Medical Sociology, Comprehensive Cancer Center Central Germany (CCCG), University Medical Center Leipzig, Philipp-Rosenthal-Str. 55, 04103 Leipzig, Germany; 2 Department of Psychosomatic Medicine and Psychotherapy, Medical Faculty, Otto-von-Guericke-University Magdeburg, Magdeburg, Germany

**Keywords:** Adolescent and young adult, Cancer, Psychooncology, Return to work, Work ability, Occupational adujustments

## Abstract

**Purpose:**

Adolescent and young adult cancer survivors (AYA-CS) face a long working life after treatment, yet factors related to a successful return to work remain largely unexplored. We therefore aimed to investigate the use of occupational adjustments and their impact on work ability upon return to work.

**Methods:**

As part of the AYA-LE study, we surveyed AYA-CS (aged 18–39 at diagnosis) who returned to work and assessed work ability (Work Ability Index) as well as use and benefit of occupational adjustments. We analyzed predictors of use and benefit of occupational adjustments on average 4 years post-diagnosis using multivariate linear and logistic regression.

**Results:**

Out of 438 AYA-CS, 389 (88.8%) returned to work after cancer diagnosis and were included in analyses. Mean work ability was M = 36.2 (SD = 6.9), 11.4% reported poor, 34.7% moderate, 41.4% good and 12.5% excellent work ability. Following treatment, 82.3% used occupational adjustments, most frequently: flexible working hours, gradual reintegration and reduced working hours. The probability of a reduction in working hours was found to be higher among older AYA-CS (≥ 30), female gender and with a fatigue index ≥ 11 (R2 = 0.073). A fatigue index < 11, elevated levels of pain and the presence of metastases/recurrence were associated with a lower benefit of reduced working hours (R2 = 0.183). Younger age (< 30) and stem cell transplant were associated with a lower benefit of support from colleagues (R2 = 0.077).

**Conclusion:**

Our results highlight the need for targeted occupational counselling throughout the treatment and even beyond the return-to-work process, considering individual and social factors.

## Introduction

When young adults are diagnosed with cancer at the beginning of their occupational career, this can lead to considerable consequences for their professional development as well as to financial disadvantages (Janssen et al. 2022). The cohort of young adults is both vulnerable and diverse, comprising individuals of varying ages and occupational statuses, including trainees, students, young professionals, and more experienced professionals. The social and financial consequences of a cancer disease affect young adults differently depending on their individual life situation (Deutsche Gesellschaft für Hämatologie und Medizinische Onkologie 2016).

The National Cancer Institute (NCI) refers to young adult cancer patients between the ages of 15 and 39 as adolescents and young adults (AYA) (Adolescent and Young Adult Oncology Progress Review Group. Closing the gap: research and care imperatives for adolescents and young adults with cancer 2006.). Due to the lengthy and intensive nature of cancer therapies, adolescent and young cancer survivors (AYA-CS) are often unable to work or study, which means they have to accept sickness absences and career breaks. Consequently, they frequently experience financial hardship (Leuteritz et al. 2021; Altherr et al. 2023; Di Giuseppe et al. 2023). The economic concerns are further exacerbated when the treatment-related physical and cognitive limitations lead to an extended reduction in working hours after treatment is completed, an occupational reorientation or when the occupation can no longer be pursued (Bhatt et al. 2021; Vizer et al. 2022).

In consideration of the overall 5-year survival rates of above 84% (Zentrum für Krebsregisterdaten, Robert-Koch-Institut.; Close et al. 2019) and their relatively young age, AYA-CS are likely to have a long working life ahead of them after treatment. This makes the successful (re)integration into the workforce a highly desirable outcome. AYA-CS are diagnosed with cancer at a stage of life in which they may have little to no work experience and are often in temporary employment situations, which can prevent AYA-CS from becoming financially independent and self-sufficient (Janssen et al. 2022). This burden can lead to family problems, increased distress, lower self-esteem and reduced quality of life (Fenn et al. 2014; Meernik et al. 2020).

Although a large proportion of AYA-CS return to work after completing treatment, they show higher unemployment rates one and five years after diagnosis, are more likely to receive disability benefits, report a lower income and have a greater need for vocational reorientation compared to age and sex matched controls (Janssen et al. 2022; Di Giuseppe et al. 2023).

The employment rate does not provide adequate information on the work ability (WA) of AYA-CS, as their ability to cope with occupational demands. Few studies investigated WA of AYA-CS, however only with single item measures, which limits their validity and reliability (König et al. 2016; Dahl et al. 2019). In their systematic review, Boelhouwer et al. report that no data were available on the course of work ability beyond the first two years following diagnosis (Boelhouwer et al. 2021). Our research group has shown that more than 75% of AYA-CS report a reduction in WA after cancer treatment completion. One year later still 57% report reduced WA (Brock et al. 2022).

It remains unclear which particular occupational adjustments (OA) AYA-CS had to use when returning to work in order to match their own occupational performance with professional demands. Treatment-related issues often require adjustments to the work situation, such as a reduction in working hours or professional reorientation (Mentschke et al. 2017). In Germany, social security agencies (including insurance providers for statutory health-, long-term care, accident, and pension coverage, as well as the employment agency) offer a wide range of services to facilitate the vocational reintegration of cancer survivors (Deutsche Gesellschaft für Hämatologie und Medizinische Onkologie 2016). Evidence demonstrates that an evaluation of these social security provisions remains an important aspect after vocational reintegration, as excessive demands frequently manifest months later (Stone et al. 2017; Boelhouwer et al. 2021). However, there is a lack of studies that investigate OA among AYA-CS and explore available social security services and their utilization. A scientific evaluation of this topic is therefore urgently needed (Deutsche Gesellschaft für Hämatologie und Medizinische Onkologie 2016; Stone et al. 2017; Heidkamp et al. 2019). This study investigates WA as well as the utilization and benefits of OA among AYA-CS in Germany after completion of cancer treatment. The following research questions were addressed:

1. What is the work ability (WA) reported by AYA-CS?
2. What occupational adjustments (OA) are used by AYA-CS?
3. How do AYA-CS evaluate the subjective benefit of these occupational adjustments?
4. Are sociodemographic, medical, or psychosocial variables associated with the utilization and the subjective benefit of certain occupational adjustments?

## Methods

### Study design

The study was part of the AYA-LE study (2014–2022) - a nationwide prospective longitudinal study with a total of six measurement points to investigate the life satisfaction, care situation and support needs of adolescents and young adults with cancer (Geue et al. 2021). Data collection for the third measurement point (t3) focusing on the employment and financial situation of AYA-CS took place from May to December 2018. The inclusion criteria for all study participants were (1) a cancer diagnosis within the previous 4 years (first manifestation, all malignant tumor entities ICD-10 C00-C97), (2) age at diagnosis between 18 and 39 years. The study received approval from the Ethics Committee of the Faculty of Medicine at the University of Leipzig (Ref. No. 436-17-13112017).

### Recruitment

The recruitment of AYA-CS was conducted in collaboration with 16 acute oncological clinics, four rehabilitation clinics, and two tumor registries. In addition, interested patients were given the opportunity to register through social media (project website and Facebook). After providing written informed consent to participate, AYA-CS received either a link to a standardized online questionnaire (LimeSurvey) or a printed version at each survey point. They were provided with an expense allowance of 10 EUR for completing each questionnaire. A detailed description of the recruitment procedure has already been published (Geue et al. 2021).

### Instruments

#### Work ability

The German version of the Work Ability Index short version (WAI-r) was used to assess work ability of AYA-CS (Hasselhorn and Freude 2007; Ilmarinen 2009). The WAI-r is an established survey instrument used in clinical and workplace health promotion and research. It shows the extent to which an employee is able to perform his or her work in view of their personal circumstances and working conditions (Zwart et al. 2002). The original questionnaire consists of seven dimensions that consider the demands of the work, the employee’s health condition and the individual resources. For example with the first item, participants were asked to rate their current WA with their lifetime best (0 = cannot work at all, to 10 = best ever). The questionnaire has been validated in various studies and the predictive power for the future course of WA is high and considered reliable with a Cronbachs α = 0.83 (Zwart et al. 2002; Bethge et al. 2012). The overall index is based on the information provided in seven dimensions and was used in the present study to evaluate WA. The WAI-r index provides a value between 7 and 49, allowing the current WA to be classified as “poor” (7–27), “moderate” (28–36), “good” (37–43), or “excellent” (44–49) (Ilmarinen 2009).

### *Questions about occupational adjustments*

Three self-developed items were used to ask participants about the OA (listed in Table 2) they used to support their return to work since completing cancer treatment (“yes”, “no”, multiple responses allowed). Participants were also asked about the benefit of the OA, i.e. how helpful these were (4-point Likert scale: 0=”not helpful”, 1=”not very helpful”, 2=”somewhat helpful”, 3=”very helpful”), and whether they were still using them currently (“yes”, “no”). The list of measures of OA, social insurance classifications, and various funding bodies was created after extensive literature research and in collaboration with an experienced social worker with relevant expertise in social law.

### *Fatigue*

The general cancer-related fatigue was measured using the EORTC QLQ-FA-12 (Weis et al. 2017). The questionnaire consists of 10 items that assess physical, cognitive, and emotional fatigue as well as two additional global items as indicators for interference of fatigue with daily activities and social sequelae of fatigue. All items can be rated on a 4-point Likert scale (1=”not at all”, 2=”a little”, 3=”moderately”, 4=”very”). Here, an overall index (items 1–10) was used with a range from 0 to 30, with higher values indicating a higher extent of fatigue based on a cutoff ≥ 11 (Friedrich et al. 2018).

### *Pain*

The German version of the Brief Pain Inventory (BPI) (Radbruch et al. 1999) is a tool for measuring pain intensity within the last 24 hours and pain-related interference with function, i.e. its effects on general emotional and functional dimensions. We used the BPI pain interference scale that is scored as the mean of the seven interference items. The questions can be answered on an 11-point Likert scale from 0=”does not interfere” to 10=”completely interferes”.

### *Sociodemographic, medical, and occupational variables*

In addition, general sociodemographic, medical, and occupational information was collected using self-developed items.

#### Statistical analysis

IBM SPSS Statistics 27 was used for statistical data analyses. Descriptive analyses were conducted to describe the sample as well as employment situation and WA.

In addition, multiple linear and logistic regression analyses were calculated to investigate which independent variables affected the utilization and subjective benefit (dependent variables) of certain OA at t3. The following predictors were selected on the basis of correlation analyses with the dependant variables (pearson correlation > 0.10) and existing literature on older cancer patients (Paltrinieri et al. 2018; Arndt et al. 2019; Boelhouwer et al. 2021):

(I) Sociodemographic variables: age, gender (male, female), relationship (yes, no).
(II) Medical variables: stem cell transplant (yes, no), metastases/recurrence (yes, no).
(III) Psychosocial variables: pain (BPI mean score 0–10), EORTC QLQ-FA12 – fatigue index cutoff (< 11, ≥ 11).

## Results

After screening for the inclusion criteria, *N* = 762 participants provided written consent and *N* = 577 completed the initial survey (t1). Between t1 and t3, the drop-out rate was 24%. In total, the data of *N* = 438 AYA-CS were available at t3.

In order to investigate the utilisation and benefits of OA, only AYA-CS who initially returned to work after a cancer diagnosis were included in the statistical analysis, regardless of their current employment status. The final sample thus consisted of *N* = 389 AYA-CS. 11% of the AYA-CS (*n* = 49) have been continuously unemployed since their diagnosis. The mean age at diagnosis was 29.9 years (SD = 6.0), and three-quarters were women (*n* = 293, 75.3%). The most common diagnoses were hematological malignancies (*n* = 121, 31.1%) and breast cancer (*n* = 100, 25.7%). Further sociodemographic, medical and psychosocial characteristics of the final sample are shown in Table 1.

**Table 1:** Sociodemographic and medical characteristics of the sample at t3 (*N* = 389)

	*N* (%)	M (SD)	Median (IQR)	
*Sociodemographic characteristics*				
Age at diagnosis		29.9 (6.0)	30.1 (24.8–35.2)	
Gender				
Male	96 (24.7)			
Female	293 (75.3)			
Highest educational degree				
No school completion/≤10 years	141 (37.2)			
> 10 years	238 (62.8)			
Relationship (yes)	294 (76.2)			
Children (yes)	161 (41.4)			
Monthly net income per household				
< 3000 EUR	193 (54.2)			
≥ 3000 EUR	163 (45.7)			
Recruitment				
Rehabilitation clinics	148 (38.0)			
Acute care hospitals	43 (11.1)			
Tumour registries	116 (29.8)			
Self-registration (e.g. homepage, e-mail)	82 (21.1)			
*Medical characteristics*				
Time since diagnosis in months		49.9 (9.7)	49.0 (42.4–55.3)	
Cancer type				
Breast cancer	100 (25.7)			
Gynecological cancer	36 (9.3)			
Testicular cancer	35 (9.0)			
Thyroid cancer	26 (6.7)			
Melanoma	14 (3.6)			
Hematological malignancies	121 (31.1)			
Sarcoma	15 (3.9)			
Gastrointestinal cancer	18 (4.6)			
Other	24 (6.2)			
Metastases or recurrence (yes)	32 (8.4)			
Therapy^a^				
Chemotherapy^b^	235 (60.4)			
Radiotherapy^c^	143 (36.8)			
Surgery	248 (63.8)			
Surgery and Chemotherapy^b^	176 (45.2)			
Surgery and Radiotherapy^c^	113 (29.0)			
Hormone therapy	101 (26.0)			
Stem cell transplant	16 (4.1)			
Follow-up medical treatment/rehabilitation (yes)	300 (77.1)			
Status of severe disability (yes)	293 (75.3)			
*Psychosocial characteristics*				
Work ability (WAI)		36.2 (6.9)	37.3 (32.0–41.0)	
Fatigue index (EORTC QLQ-FA-12)		11.6 (7.4)	11.0 (5.0–18.0)	
Pain interference scale (BPI)		1.1 (1.6)	0.3 (0.0-1.7)	

Percentages may not add up to 100% due to missing data, M = Mean, SD = standard deviation, IQR = interquartile range, WAI Work Ability Index (scale 7–49), EORTC QLQ-FA-12 mean fatigue index (scale 0–30), BPI Brief Pain Inventory mean pain interference scale (scale 0–10)

a = multiple answers possible

b = includes radiochemotherapy

c = includes radiochemotherapy and nuclear therapy

### Employment

At the third measurement point, almost 87% (*n* = 338) of the AYA-CS who had ever returned to work since the cancer diagnosis were currently employed and 10% (*n* = 38) were studying or in training. Almost two-thirds (61%, *n* = 236) were working full-time (again) and about a quarter (26%, *n* = 102) of AYA-CS reported reduced working hours. A large proportion of AYA-CS (60%, *n* = 232) reported primarily executive jobs with decision-making responsibilities. 17% (*n* = 66) worked in an executive capacity under supervision and 11% (*n* = 43) reported high levels of responsibility at work in managerial or executive positions. Over 60% (*n* = 243) of AYA-CS were engaged in predominantly mental or cognitive work, while the employment of 24% (*n* = 92) involved predominantly manual or physical work.

A quarter of the AYA-CS (24%, *n* = 92) stated that the cancer disease forced them to work more slowly or to change their working method. About 32% (*n* = 123) reported being able to do the work but with discomfort, and 7% (*n* = 28) were incapacitated for work. In addition, half of the AYA-CS (50%, *n* = 198) indicated that they had applied for a new job since their cancer diagnosis (42% were successful, *n* = 165) and 32% (*n* = 124) reported that their financial situation had worsened due to their cancer disease.

### Work ability

Mean WA was M = 36.2 (SD = 6.9). In detail, 11.4% of the AYA-CS reported poor, 34.7% moderate, 41.4% good and 12.5% excellent WA. A large proportion (66%, *N* = 256) reported being more psychologically than physically challenged by the work.

### Utilization and subjective benefit of occupational adjustments

Table 2 displays the frequencies of AYA-CS using OA and the mean subjective benefit (scale 0–3), respectively.

**Table 2:** Utilization and subjective benefit of occupational adjustments (*N* = 389)

	ever used *N* (%)	current utilization *N* (%)	benefit M (SD)
Reduction of weekly working hours	122 (31.4)	58 (14.9)	2.71 (0.5)
Flexible working hours	138 (35.5)	97 (24.9)	2.87 (0.4)
Leave of absence from work/training (e.g. semester on leave, sabbatical etc.)	51 (13.1)	10 (2.6)	2.79 (0.5)
Postponement of examinations relevant to training	44 (11.3)	10 (2.6)	2.70 (0.6)
Different/fewer work tasks to reduce physical burden	69 (17.7)	25 (6.4)	2.60 (0.6)
Different/fewer work tasks to reduce psychological burden	72 (18.5)	41 (10.5)	2.70 (0.5)
Changes of equipment at the workplace/use of aids	42 (10.8)	27 (6.9)	2.57 (0.6)
Support from colleagues	115 (29.6)	44 (11.3)	2.62 (0.6)
Individual arrangements	77 (19.8)	31 (8.0)	2.92 (0.3)
Home office/mobile working	11 (2.8)		
Exemption from/adjustment of roster	25 (6.4)		
Exemption from certain activities	8 (2.1)		
Compensation for disadvantage in exams	2 (0.5)		
Other	6 (1.5)		
Measures of occupational reintegration	169 (43.4)	1 (0.3)	2.62 (0.6)
via statutory pension insurance	61 (15.7)		
via statutory health insurance	82 (21.1)		
Gradual reintegration („Hamburger Modell“^a^)	125 (32.1)		
Cushioning due to remaining leave or overtime	6 (1.5)		
Other	42 (10.8)		
Occupational integration management (“BEM”^b^)	22 (5.7)	7 (1.8)	2.18 (1.1)
Measures for workplace health promotion	26 (6.7)	17 (4.4)	2.31 (0.7)
Measures by representative body for disabled employees	34 (8.7)	20 (5.1)	2.34 (0.9)
Measures by works or staff council	23 (5.9)	7 (1.8)	2.24 (0.9)
Measures for promoting occupational participation	17 (4.4)	5 (1.3)	2.80 (0.4)
Other	7 (1.8)	3 (0.8)	2.60 (0.9)

Benefit = mean and standard deviation for the item „How helpful was this occupational adjustment for you?“, scale from 0 = not helpful to 3 = very helpful

a = a rehabilitation measure involving a step-by-step approach of reintegration into the workplace with reduced hours and under protective conditions (in German calles “Hamburger Modell”)

b = individual assistance in returning to work after a longer period of illness (in German called “Betriebliches Eingliederungsmanagement”)

### Predictors for the utilization of occupational adjustments

A total of *N* = 320 (82.3%) AYA-CS had used OA since their cancer diagnosis. In the regression analyses, utilization and subjective benefit of the four OA with the highest utilization rates (ever, not current) were considered as dependent variables: (I) reduction in working hours, (II) flexible working hours, (III) support from colleagues, (IV) gradual reintegration.

An age ≥ 30, female gender, and a fatigue index ≥ 11 were associated with a higher probability of utilizing a reduction in working hours (Table 3), with a model fit of Nagelkerke *R*^*2*^ = 0.073 (Muijs 2011). The model’s prediction ability was statistically significant. We found no significant associations between sociodemographic, medical, or psychosocial variables and the utilization of flexible working hours, support from colleagues or gradual reintegration. Further results of the regression analyses are listed in Table 3.

**Table 3:** Results of multiple logistic regression analyses for utilization of occupational adjustments

	Reduction in working hours	Flexible working hours	Support from colleagues	Gradual reintegration
	*N* = 354, *R*^*2*^ = 0.073	*N* = 353, *R*^*2*^ = 0.049	*N* = 352, *R*^*2*^ = 0.055	*N* = 352, *R*^*2*^ = 0.034
*χ²-statistic*	*χ²*(7) = 19.124	*χ²*(7) = 12.855	*χ²*(7) = 13.983	*χ²*(7) = 9.122
*p-value*	***p =*** **.008**	*p* =.076	*p* =.051	*p* =.244
	OR (*p*-value)			
*Sociodemographic*				
Age^a^	**1.05 (*p*** **=.026)**	1.02 (*p* =.352)	0.99 (*p* =.518)	1.02 (*p* =.199)
Gender^b^	**2.03 (*p*** **=.016)**	2.36 (*p* =.002)	1.57 (*p* =.126)	1.41 (*p* =.184)
Relationship^c^	1.13 (*p* =.657)	0.94 (*p* =.825)	1.01 (*p* =.972)	1.12 (*p* =.664)
*Medical*				
Stem cell transplant^d^	1.11 (*p* =.858)	1.69 (*p* =.369)	1.63 (*p* =.387)	0.56 (*p* =.327)
Metastases/recurrence^e^	0.92 (*p* =.752)	0.79 (*p* =.359)	1.00 (*p* =.993)	1.27 (*p* =.316)
*Psychosocial*				
Pain (BPI)	0.89 (*p* =.180)	1.02 (*p* =.848)	1.05 (*p* =.524)	0.99 (*p* =.900)
Fatigue (EORTC)^f^	**1.82 (*p*** **=.022)**	1.01 (*p* =.972)	1.90 (*p* =.015)	1.32 (*p* =.251)

*R*^*2*^ = Nagelkerke *R*^*2*^, *X*^*2*^ = Chi Square Statistic, OR = Odds Ratio, *p* = type-I-error probability, significance level *p* <.05 = bold type, response categories: a = ≥ 30 years, b = female, c = in a relationship, d = yes, e = yes, f = EORTC QLQ-FA-12 fatigue-index > 11

### Predictors for the subjective benefit of occupational adjustments

A fatigue index ≥ 11 was associated with a higher subjectively rated benefit of a reduction in working hours (Table 4). Higher pain impairment and the presence of metastases or recurrence were associated with a lower subjectively rated benefit of a reduction in working hours (Table 4). 18% of the variance in subjective benefit of the reduction in working hours could be explained by the regression model (adjusted *R*^*2*^ = 0.183). The model’s prediction ability was statistically significant. An age ≥ 30 years was also associated with a subjectively higher rated benefit of support from colleagues, while a stem cell transplant was negatively associated. The model fit is *R*^2^ = 0.077.

We found no significant associations between sociodemographic, medical, or psychosocial variables and the subjective benefit of flexible working hours or gradual reintegration. Further results of the regression analyses are listed in Table 4.

**Table 4:** Multiple linear regression analyses for subjective benefit of occupational adjustments

	Reduction in working hours	Flexible working hours	Support from colleagues	Gradual reintegration
	*N* = 116, *R*^*2*^ = 0.183	*N* = 129, *R*^*2*^=-0.024	*N* = 105, *R*^*2*^ = 0.077	*N* = 160, *R*^*2*^ = 0.004
*F-statistic*	F(7, 108) = 4.688	F(7, 121) = 0.578	F(7, 97) = 2.234	F(7, 152) = 1.103
*p-value*	**< 0.001**	0.773	**0.038**	0.364
	Beta *β* (*p*-value)			
*Sociodemographic*				
Age^a^	− 0.085 (*p* =.339)	0.047 (*p* =.619)	**0.206 (*p*** **=.042)**	− 0.118 (*p* =.151)
Gender^b^	0.148 (*p* =.105)	0.122 (*p* =.205)	− 0.170 (*p* =.093)	0.049 (*p* =.555)
Relationship^c^	− 0.052 (*p* =.567)	− 0.098 (*p* =.291)	0.033 (*p* =.737)	− 0.065 (*p* =.434)
*Medical*				
Stem cell transplant^d^	0.106 (*p* =.250)	0.003 (*p* =.976)	**− 0.206 (*p*** **=.039)**	− 0.042 (*p* =.609)
Metastases/recurrence ^e^	**− 0.243 (*p*** **=.006)**	0.078 (*p* =.393)	0.041 (*p* =.672)	0.023 (*p* =.780)
*Psychosocial*				
Pain (BPI)	**− 0.316 (*p*** **=.001)**	0.011 (*p* =.920)	0.107 (*p* =.328)	− 0.160 (*p* =.076)
Fatigue (EORTC)^f^	**0.253 (*p*** **=.015)**	− 0.095 (*p* =.380)	− 0.107 (*p* =.322)	0.004 (*p* =.963)

*R*^*2*^ = adjusted *R*^*2*^, *β* = standardized beta coefficient, *p* = type-I-error probability, significance level *p* <.05 = bold type, response categories: a = ≥ 30 years, b = female, c = in a relationship, d = yes, e = yes, f = EORTC QLQ-FA-12 fatigue-index > 11

## Discussion

This study aimed to investigate the work ability of AYA-CS in Germany and to assess how they utilize and benefit from occupational adjustments.

### Work ability

On average four years after diagnosis (t3), the majority of AYA-CS (87%) who had ever returned to work since the cancer diagnosis were currently employed. However, more than 46% reported reduced WA with a Work Ability Index in the „poor“ to „moderate“ range.

Merely considering employment rates is therefore not an indicator of the ability to cope with occupational demands. Currently, there is a lack of studies assessing the WA of (AYA) cancer patients using the complete Work Ability Index, which makes comparisons difficult to draw at this point. With regard to other results of the AYA-LE study, that have already been published (Brock et al. 2022), it was found that more than 75% of AYA-CS reported reduced WA after cancer treatment (t1) and 57% one year later (t2). Therefore, the percentage of AYA-CS with reduced WA appears to decline over time. Dahl et al. found lower (38%) frequency rates with reduced WA, but data collection was 16 years after diagnosis (Dahl et al. 2019). However, both studies used assessed the WA of AYA-CS using single-item measures. While current norm data are unfortunately not available, a comparison with the same aged general population based on older normative values from a German reference dataset ranked the mean WA of AYA-CS in the 25%-percentile (Hasselhorn and Freude 2007). Similarly in this study, 75% of the same aged general population reported a higher Work Ability Index than AYA-CS, which indicates that on average AYA-CS do not achieve age-appropriate WA even four years after diagnosis. Furthermore, the WA of AYA-CS in this study was possibly even overestimated as AYA-CS who had not worked since diagnosis were not included in data analysis. Unemployed AYA-CS had often undergone systemic treatments, and heavier treatment burdens may affect WA even more profoundly (Janssen et al. 2022). Thus, in the context of long-term care, it is not only important to ensure that AYA-CS return to work after successful treatment, but also to consider how they return to work, i.e. providing long-term support to improve WA of AYA-CS.

### Utilization of occupational adjustments

Regarding the utilization of OA, the results indicate that more than 82% of AYA-CS have made OA since their cancer diagnosis. This suggests that returning to work without limitations is rarely feasible for the majority of AYA-CS. Unfortunately, there are no comparable studies available specifically for the AYA age group. However, a recent investigation conducted on older breast cancer patients in Germany revealed that 57% of respondents reported at least one vocational change since their diagnosis. The utilization of OA appears to be lower among older cancer patients, potentially indicating a higher prevalence of long-term consequences and resulting workplace limitations among AYA-CS. Several studies have already demonstrated that AYA-CS experience a greater psychological burden and face an increased risk of long-term effects compared to older individuals, possibly due to intensive treatments (Bifulco et al. 2012; Shay et al. 2016). The higher utilization of OA in this group may be reflective of these factors.

The majority of AYA-CS in the present study appear to be aware of and utilize the corresponding adjustments; however, considering the average WA, these adjustments do not seem to create a sufficient alignment between the occupational demands and the (work) performance of AYA-CS. The most frequently used vocational adjustments were gradual reintegration, flexible working hours, and a reduction in working hours. In addition to structural adjustments, the support provided by colleagues was of great relevance. This support cannot be attributed to a specific form of social legal assistance and may be of both practical and social significance.

Higher age (≥ 30 years), female gender, and higher fatigue symptomatology (fatigue index ≥ 11) were associated with a higher likelihood of utilizing a reduction in working hours. The higher utilization of part-time work from the age of 30 may, on the one hand, be related to the fact that many AYA-CS in this age group have children and therefore prefer to reduce their weekly hours. This applies particularly to women who tend to take on more childcare responsibilities than men. Another explanation may be that for AYA-CS at a later stage of life a reduction in weekly working hours entails lower financial losses than those experienced by younger AYA-CS just entering the workforce. The latter group often works under precarious employment conditions and may not be able to financially afford part-time work, particularly after a cancer diagnosis.

### Benefit of occupational adjustments

The subjective benefit of the individual OA ranged on average from “a little helpful” to “very helpful”. AYA-CS attributed the greatest subjective benefit to the following occupational adjustments: individual arrangements, measures for promoting occupational participation, flexible working hours, leave of absence from work or training.

In particular, AYA-CS with high levels of fatigue (fatigue index ≥ 11) seem to benefit from a reduction in weekly working hours, presumably due to the reduced workload. However, AYA-CS experiencing severe pain, metastases, or recurrence report the least benefit from a reduction in weekly working hours. The physical and psychological limitations (e.g., pain, fatigue, fear of progression) may be so burdensome for these individuals that part-time work does not seem to provide added value. Other forms of support, such as pain management and psycho-oncological care, may be more effective in this case.

Furthermore, AYA-CS who reported that they benefited less from support provided by colleagues were younger (< 30 years) or had undergone a stem cell transplantation. Younger AYA-CS, especially those with hematological neoplasms who often undergo stem cell transplantation, experience particularly long periods of (social) isolation and work disruption. They report more distressing social interactions within their support network compared to older cancer patients with the same diagnosis, which, in turn, has a stronger impact on their quality of life (Geue et al. 2019). It may be challenging for colleagues to accommodate the prolonged work absence of AYA-CS who have undergone stem cell transplantation. Structural measures seem to be primarily required in these cases.

As only a few specific variables could be identified that show particular benefit for specific adjustments (with little to moderate effects) and as all OA were rated as helpful on average, they seem to be of general value for AYA-CS. Furthermore, the benefit seems to depend less on the cancer diagnosis, but rather on symptom-related and individual factors.

### Limitations

The results of this study must be interpreted in light of several limitations. Higher education levels and female gender were overrepresented in the present sample, which is a common phenomenon in clinical trials (Wakefield et al. 2017). It should be noted that AYA-CS from lower socioeconomic backgrounds often work in jobs with less flexible structures, thereby potentially experiencing even greater financial losses and constraints following a cancer diagnosis. Additionally, over 75% of the AYA-CS in this study were in a partnership at the time of the survey. The negative occupational and financial effects of cancer and its treatment on single or sole-parenting AYA-CS may potentially be even more pronounced. Furthermore, patients who did not return to work were not analyzed in the present study. Their WA is probably more limited and they may also have used OA since diagnosis, which were not considered.

### Research and clinical implications

Regarding WA of AYA-CS, future research should consider comparisons with healthy peers, as the normative data of the 2007 Work Ability Index (Hasselhorn and Freude 2007) are already quite outdated. Moreover, clear gaps in the literature need to be addressed in order to better understand the occupational situation of AYA-CS with low socio-economic status, male gender, and migrant background, thereby mapping underrepresented groups in research (Butow et al. 2020). Furthermore, it is important to examine how interventions and AYA-CS survivorship programmes should be designed to optimally prepare them for their return to work, to achieve maximum alignment between job requirements and WA and to provide age-appropriate long-term support. Initial efforts to address this endeavor can already be observed internationally (Adams et al. 2021; Janssen et al. 2021). AYA-CS need to be fully and comprehensively informed about existing entitlements and access routes in order to realize their social rights. This requires low-threshold and individual social legal counseling during treatment, as well as long-term support during aftercare. Strauser et al. found that the utilization of job search assistance and on-the-job support was associated with a fourfold increased probability of being employed among AYA-CS (Strauser et al. 2010). Currently, a Germany-wide feasibility study (CARES) is investigating the intensified and needs-oriented support of cancer survivors in their return to work through vocational guides. This type of vocational support can help reduce or avoid interruptions in aftercare that are caused by constantly changing contacts. Often, AYA-CS have to repeatedly seek contact with supporting individuals and institutions, reformulate their issues, and rebuild relationships. It can be assumed that AYA-CS risk groups, in particular individuals with migrant backgrounds or language barriers, and also men who are less likely to use support services, face even greater difficulties in accessing support within these structures (Janßen et al. 2021).

Furthermore, it is important to address individual and structural barriers in clinical practice. In the present study, 75% of AYA-CS have a disability status, but only 9% utilize measures provided by the representative body of severely disabled employees. This imbalance may potentially be attributed to stigmatization, as AYA-CS may fear workplace disadvantages due to their additional disability status. Additionally, young adults, who had often just entered the job market, are in dependent professional positions and do not want to burden employers and colleagues upon their return to work after long periods of absence. Their goal is to close financial gaps and to no longer be (considered) ill, which reduces the utilization of OA. Similar barriers have been previously identified with regard to the utilization of rehabilitation (Heidkamp et al. 2019).

In order to facilitate a long-term successful return to work for AYA-CS, multiple stakeholders need to collaborate: AYA-CS themselves, occupational health care providers, employers, as well as rehabilitation and social insurance institutions (Boer et al. 2015). In addition to multiprofessional collaboration, the use of multifactorial interventions is essential (Butow et al. 2020). These should focus on survivors’ goals, social legal information including OA, enhancing WA, stress management, cognitive skills training and dealing with financial losses. The realization of these interventions is best achieved through the establishment of widespread and comprehensive cancer survivorship care programmes for AYA-CS.

## Conclusion

Achieving maximum work ability and returning to work without restrictions remains challenging for AYA-CS. Targeted occupational counselling in the course of the disease and even beyond the return-to-work process, taking into account individual and social factors, may help AYA-CS minimize (work) limitations. In order to compensate for disadvantages it is important to adapt the workplace and the work situation to AYA-CS special needs. They are among the most productive members of society, but they need to be well supported and informed about their opportunities, which can be best realised through multimodal and personalized cancer survivorship programmes. Enabling a professional career despite cancer should therefore be a major goal for the aftercare of AYA-CS.

## Data Availability

No datasets were generated or analysed during the current study.

## Abbreviations

AYA: Adolescents and young adults
AYA-CS: Adolescent and young adult cancer survivors
WA: Work ability
OA: Occupational adjustments

## References

[CR1] Adams SC, Herman J, Lega IC, Mitchell L, Hodgson D, Edelstein K, Travis LB, Sabiston CM, Thavendiranathan P, Gupta AA (2021) Young Adult Cancer Survivorship: recommendations for patient Follow-up, Exercise Therapy, and Research. JNCI Cancer Spectr 5. 10.1093/jncics/pkaa099 10.1093/jncics/pkaa099 PMC7919337 33681702

[CR2] Altherr A, Bolliger C, Kaufmann M, Dyntar D, Scheinemann K, Michel G, Mader L, Roser K (2023) Education, Employment, and Financial outcomes in adolescent and young Adult Cancer Survivors—A systematic review. Curr Oncol 30:8720–8762. 10.3390/curroncol30100631 37887531 10.3390/curroncol30100631 PMC10604989

[CR3] Arndt V, Koch-Gallenkamp L, Bertram H, Eberle A, Holleczek B, Pritzkuleit R, Waldeyer-Sauerland M, Waldmann A, Zeissig SR, Doege D, Thong MSY, Brenner H (2019) Return to work after cancer. A multi-regional population-based study from Germany. Acta Oncol 58:811–818. 10.1080/0284186X.2018.1557341 30777496 10.1080/0284186X.2018.1557341

[CR4] Bethge M, Radoschewski FM, Gutenbrunner C (2012) The work ability index as a screening tool to identify the need for rehabilitation: longitudinal findings from the second German Sociomedical Panel of Employees. J Rehabil Med 44:980–987. 10.2340/16501977-1063 23027375 10.2340/16501977-1063

[CR5] Bhatt NS, Brazauskas R, Salit RB, Syrjala K, Bo-Subait S, Tecca H, Badawy SM, Baker KS, Beitinjaneh A, Bejanyan N, Byrne M, Dias A, Farhadfar N, Freytes CO, Ganguly S, Hashmi S, Hayashi RJ, Hong S, Inamoto Y, Jamani K, Kasow KA, Khera N, Krem MM, Lazarus HM, Lee CJ, Lee S, Majhail NS, Malone AK, Marks DI, Mau L-W, Mayo SJ, Muffly LS, Nathan S, Nishihori T, Page KM, Preussler J, Rangarajan HG, Rotz SJ, Salooja N, Savani BN, Schears R, Schechter-Finkelstein T, Schiller G, Shah AJ, Sharma A, Wang T, Wirk B, Battiwalla M, Schoemans H, Hamilton B, Buchbinder D, Phelan R, Shaw B (2021) Return to work among Young Adult survivors of Allogeneic Hematopoietic Cell Transplantation in the United States. Transpl Cell Ther 27:679. e1-679.e8 10.1016/j.jtct.2021.04.013 PMC8425287 33895402

[CR6] Bifulco G, de Rosa N, Tornesello ML, Piccoli R, Bertrando A, Lavitola G, Morra I, Di Spiezio Sardo A, Buonaguro FM, Nappi C (2012) Quality of life, lifestyle behavior and employment experience: a comparison between young and midlife survivors of gynecology early stage cancers. Gynecol Oncol 124:444–451. 10.1016/j.ygyno.2011.11.033 22119994 10.1016/j.ygyno.2011.11.033

[CR7] Boelhouwer IG, Vermeer W, van Vuuren T (2021) The associations between late effects of cancer treatment, work ability and job resources: a systematic review. Int Arch Occup Environ Health 94:147–189. 10.1007/s00420-020-01567-w 32929528 10.1007/s00420-020-01567-w PMC7873002

[CR9] Brock H, Friedrich M, Sender A, Richter D, Geue K, Mehnert-Theuerkauf A, Leuteritz K (2022) Work ability and cognitive impairments in young adult cancer patients: associated factors and changes over time-results from the AYA-Leipzig study. J Cancer Surviv 16:771–780. 10.1007/s11764-021-01071-1 34118000 10.1007/s11764-021-01071-1 PMC9300567

[CR10] Butow P, Laidsaar-Powell R, Konings S, Lim CYS, Koczwara B (2020) Return to work after a cancer diagnosis: a meta-review of reviews and a meta-synthesis of recent qualitative studies. J Cancer Surviv 14:114–134. 10.1007/s11764-019-00828-z 31858379 10.1007/s11764-019-00828-z

[CR11] Close AG, Dreyzin A, Miller KD, Seynnaeve BKN, Rapkin LB (2019) Adolescent and young adult oncology-past, present, and future. CA Cancer J Clin 69:485–496. 10.3322/caac.21585 31594027 10.3322/caac.21585

[CR12] Dahl AA, Fosså SD, Lie HC, Loge JH, Reinertsen KV, Ruud E, Kiserud CE (2019) Employment status and work ability in Long-Term Young Adult Cancer survivors. J Adolesc Young Adult Oncol 8:304–311. 10.1089/jayao.2018.0109 30900929 10.1089/jayao.2018.0109

[CR8] de Boer AGEM, Taskila TK, Tamminga SJ, Feuerstein M, Frings-Dresen MHW, Verbeek JH (2015) Interventions to enhance return-to-work for cancer patients. Cochrane Database Syst Rev 2015:CD007569. 10.1002/14651858.CD007569.pub3 26405010 10.1002/14651858.CD007569.pub3 PMC6483290

[CR37] de Zwart BCH, Frings-Dresen MHW, van Duivenbooden JC (2002) Test-retest reliability of the work ability index questionnaire. Occup Med (Lond) 52:177–181. 10.1093/occmed/52.4.177 12091582 10.1093/occmed/52.4.177

[CR13] Di Giuseppe G, Pagalan L, Jetha A, Pechlivanoglou P, Pole JD (2023) Financial toxicity among adolescent and young adult cancer survivors: a systematic review of educational attainment, employment, and income. Crit Rev Oncol Hematol 183:103914. 10.1016/j.critrevonc.2023.103914 36706969 10.1016/j.critrevonc.2023.103914

[CR14] Fenn KM, Evans SB, McCorkle R, DiGiovanna MP, Pusztai L, Sanft T, Hofstatter EW, Killelea BK, Knobf MT, Lannin DR, Abu-Khalaf M, Horowitz NR, Chagpar AB (2014) Impact of financial burden of cancer on survivors’ quality of life. J Oncol Pract 10:332–338. 10.1200/JOP.2013.001322 24865220 10.1200/JOP.2013.001322

[CR15] Friedrich M, Nowe E, Hofmeister D, Kuhnt S, Leuteritz K, Sender A, Stöbel-Richter Y, Geue K (2018) Psychometric properties of the fatigue questionnaire EORTC QLQ-FA12 and proposal of a cut-off value for young adults with cancer. Health Qual Life Outcomes 16:125. 10.1186/s12955-018-0949-0 29903021 10.1186/s12955-018-0949-0 PMC6002999

[CR16] Geue K, Götze H, Friedrich M, Leuteritz K, Mehnert-Theuerkauf A, Sender A, Stöbel-Richter Y, Köhler N (2019) Perceived social support and associations with health-related quality of life in young versus older adult patients with haematological malignancies. Health Qual Life Outcomes 17:145. 10.1186/s12955-019-1202-1 31438983 10.1186/s12955-019-1202-1 PMC6704656

[CR17] Geue K, Mehnert-Theuerkauf A, Stroske I, Brock H, Friedrich M, Leuteritz K (2021) Psychosocial Long-Term effects of Young Adult Cancer survivors: Study Protocol of the longitudinal AYA-LE Long-Term effects Study. Front Psychol 12. 10.3389/fpsyg.2021.688142 10.3389/fpsyg.2021.688142 PMC8511386 34659005

[CR18] Hasselhorn H-M, Freude G (2007) Der Work-Ability-Index: Ein Leitfaden. Schriftenreihe Der Bundesanstalt für Arbeitsschutz Und Arbeitsmedizin Sonderschrift, S 87. Wirtschaftsverl. NW Verl. für neue Wiss, Bremerhaven

[CR19] Heidkamp P, Hiltrop K, Kowalski C, Ernstmann N (2019) Berufliche Wiedereingliederung Nach Brustkrebs – Vorstellung Der B–CARE-Studie. Forum 34:57–59. 10.1007/s12312-018-0547-4

[CR20] Ilmarinen J (2009) Work ability–a comprehensive concept for occupational health research and prevention. Scand J Work Environ Health 35:1–5. 10.5271/sjweh.1304 19277432 10.5271/sjweh.1304

[CR22] Janssen SHM, van der Graaf WTA, van der Meer DJ, Manten-Horst E, Husson O (2021) Adolescent and young adult (AYA) Cancer Survivorship practices: an overview. Cancers (Basel) 13. 10.3390/cancers13194847 10.3390/cancers13194847 PMC8508173 34638332

[CR23] Janssen SHM, van der Meer DJ, van Eenbergen MCHJ, Manten-Horst E, van der Graaf WTA, Husson O (2022) Short- and long-term impact of cancer on employment and financial outcomes of adolescents and young adults (AYAs): a large population-based case-control registry study in the Netherlands. ESMO Open 7:100521. 10.1016/j.esmoop.2022.100521 35772237 10.1016/j.esmoop.2022.100521 PMC9434129

[CR21] Janßen A, Schneider S, Stäudle J, Walther J (2021) Probleme Der Beruflichen (Re–)Integration Von Krebserkrankten: Wie können Wir unterstützen? (the problems of career (re)integration faced by cancer patients). Onkologe (Berl) 27:802–808. 10.1007/s00761-021-00973-0 34177129 10.1007/s00761-021-00973-0 PMC8212578

[CR24] König V, Krauth KA, Schulte T (2016) Wiedereingliederung Junger Erwachsener in die Arbeitswelt Nach Krebsbehandlung. Forum 31:315–319. 10.1007/s12312-016-0106-9

[CR25] Leuteritz K, Friedrich M, Sender A, Richter D, Mehnert-Theuerkauf A, Sauter S, Geue K (2021) Return to Work and Employment Situation of Young Adult Cancer survivors: results from the adolescent and young adult-Leipzig Study. J Adolesc Young Adult Oncol 10:226–233. 10.1089/jayao.2020.0055 32746763 10.1089/jayao.2020.0055

[CR26] Meernik C, Kirchhoff AC, Anderson C, Edwards TP, Deal AM, Baggett CD, Kushi LH, Chao CR, Nichols HB (2020) Material and psychological financial hardship related to employment disruption among female adolescent and young adult cancer survivors. Cancer 127:137–148. 10.1002/cncr.33190 33043464 10.1002/cncr.33190 PMC7736150

[CR38] Mentschke L, Leuteritz K, Daneck L, Breuer N, Sender A, Friedrich M, Nowe E, Stöbel-Richter Y, Geue K (2017) Krebs und Karriere? – Eine qualitative Untersuchung zur beruflichen Situation und Integration junger Erwachsener mit Krebs (Cancer and Career? - A Qualitative Study of Job Status of Young Adult Cancer Survivors). Psychother Psychosom Med Psychol 67:76–82. 10.1055/s-0042-122712 10.1055/s-0042-122712 28288497

[CR27] Muijs D (2011) Doing Quantitative Research in Education with SPSS, 2nd edn. SAGE, Los Angeles, CA, London, New Delhi, Singapore, Washington D.C.

[CR28] Paltrinieri S, Fugazzaro S, Bertozzi L, Bassi MC, Pellegrini M, Vicentini M, Mazzini E, Costi S (2018) Return to work in European Cancer survivors: a systematic review. Support Care Cancer 26:2983–2994. 10.1007/s00520-018-4270-6) 29845421 10.1007/s00520-018-4270-6

[CR29] Radbruch L, Loick G, Kiencke P, Lindena G, Sabatowski R, Grond S, Lehmann KA, Cleeland CS (1999) Validation of the German version of the brief Pain Inventory. J Pain Symptom Manage 18:180–187. 10.1016/S0885-3924(99)00064-0 10517039 10.1016/s0885-3924(99)00064-0

[CR30] Shay LA, Carpentier MY, Vernon SW (2016) Prevalence and correlates of fear of recurrence among adolescent and young adult versus older adult post-treatment cancer survivors. Support Care Cancer 24:4689–4696. 10.1007/s00520-016-3317-9 27387913 10.1007/s00520-016-3317-9 PMC5033688

[CR31] Stone DS, Ganz PA, Pavlish C, Robbins WA (2017) Young adult cancer survivors and work: a systematic review. J Cancer Surviv 11:765–781. 10.1007/s11764-017-0614-3 28478587 10.1007/s11764-017-0614-3 PMC5671547

[CR32] Strauser D, Feuerstein M, Chan F, Arango J, da Silva Cardoso E, Chiu C-Y (2010) Vocational services associated with competitive employment in 18–25 year old cancer survivors. J Cancer Surviv 4:179–186. 10.1007/s11764-010-0119-9 20373043 10.1007/s11764-010-0119-9

[CR33] Vizer LM, Mikles SP, Piepmeier AT (2022) Cancer-related cognitive impairment in survivors of adolescent and young adult non-central nervous system cancer: a scoping review. Psychooncology 31:1275–1285. 10.1002/pon.5980 35726379 10.1002/pon.5980

[CR34] Wakefield CE, Fardell JE, Doolan EL, Aaronson NK, Jacobsen PB, Cohn RJ, King M (2017) Participation in psychosocial oncology and quality-of-life research: a systematic review. Lancet Oncol 18:e153–e165. 10.1016/s1470-2045(17)30100-6 28271870 10.1016/S1470-2045(17)30100-6

[CR35] Weis J, Tomaszewski KA, Hammerlid E, Ignacio Arraras J, Conroy T, Lanceley A, Schmidt H, Wirtz M, Singer S, Pinto M, Alm El-Din M, Compter I, Holzner B, Hofmeister D, Chie W-C, Czeladzki M, Harle A, Jones L, Ritter S, Flechtner H-H, Bottomley A (2017) International Psychometric Validation of an EORTC Quality of Life Module Measuring Cancer related fatigue (EORTC QLQ-FA12). J Natl Cancer Inst 109. 10.1093/jnci/djw273 10.1093/jnci/djw273 28376231

[CR36] Zentrum für Krebsregisterdaten, Robert-Koch-Institut Datenbankabfrage: Überleben, Absolute Rate in Prozent in Deutschland (Gewählte Filter: Altersgruppen: 15–44; Diagnose: Krebs gesamt (C00-C97 ohne C44); Geschlecht: männlich, weiblich; Intervall-Länge in Jahren: 10; Jahre: 2013–2014). 29-11-2017. Eingesehen: 7-5-2018. https://www.krebsdaten.de/Krebs/SiteGlobals/Forms/Datenbankabfrage/datenbankabfrage_stufe2_form.html

